# Office design, telework from home, and self-certified sickness absence: A cross-sectional study of main and moderating effects in a nationally representative sample

**DOI:** 10.5271/sjweh.4078

**Published:** 2023-03-30

**Authors:** Randi Hovden Borge, Håkon A Johannessen, Knut Inge Fostervold, Morten Birkeland Nielsen

**Affiliations:** 1 Department of work psychology and physiology, National Institute of Occupational Health, Oslo, Norway; 2 Department of Psychology, University of Oslo, Oslo, Norway; 3 Department of Psychosocial Science, University of Bergen, Bergen, Norway

**Keywords:** absenteeism, health, home office, non-territorial office, open-plan office, private office, remote work, shared-room office, time-spatial flexibility

## Abstract

**Objectives:**

This study aimed to investigate (i) the main effects of office design and access to telework from home (TWFH) on self-certified sickness absence and (ii) the moderating effects of access to TWFH on the relationship between office design and self-certified sickness absence.

**Methods:**

The study used cross-sectional survey data from a nationally representative sample from Norway (N=4329). Research objectives were investigated with negative binomial hurdle models, adjusting for age, gender, education level, leadership responsibility, and time spent on office work. Moderating effects of TWFH were evaluated with pairwise comparisons and plots of estimated marginal means.

**Results:**

In adjusted models, employees in conventional open-plan offices [odds ratio (OR) 1.32, 95% confidence interval (CI) 1.13–1.54] had significantly higher odds of sickness absence than employees in private offices. Employees with access to TWFH (OR 0.86, 95% CI 0.74–0.99) had significantly lower odds of sickness absence than employees with no access. Among employees with access to TWFH, those in conventional open-plan offices had significantly higher predicted probability of self-certified sickness absence than those in private offices (*z*=4.41, P<0.0001). There were no significant differences between office designs among employees who did not have access to TWFH. There were no significant main or moderating effects on the number of sickness absence episodes in adjusted models.

**Conclusions:**

The current study identifies conventional open-plan offices as a potential risk factor for sickness absence. While access to TWFH may be a protective factor overall, it amplified – rather than attenuated – differences in sickness absence between employees in private offices and conventional open-plan offices.

Contemporary office work is constantly changing and includes a multitude of office concepts, from private offices to open-plan workspaces, and increasingly also various flexible work arrangements, such as non-territorial designs (eg, shared workstations, activity-based offices) and telework from home (TWFH) (1). This development has mainly been technology-driven (1), financially motivated (2), and accelerated by COVID-19 measures (3), rather than based on scientific knowledge about advantages and disadvantages of different concepts. One reason for this lack of knowledge translation is that research is lagging behind on how these different ways of organizing office work influence employee health and well-being (2, 4–7). While office work is changing along several dimensions, such as office design (eg, private versus open-plan offices), office use (eg, fixed versus shared seating), and office location (eg, at work versus at home) (1), most research have been limited to studying office design in isolation (4). Synthesized evidence from this research stream suggests differences in experiences of health and well-being across office designs (2, 4) and unfavorable health outcomes – such as increased risk of sickness absence (8) and disability retirement (9) – for employees in shared -room and open-plan offices. Yet, these observed differences are likely not a product of office design alone but contingent on other aspects of contemporary office work. For instance, as office work at the employer’s worksite and at home become intertwined due to increased access to TWFH (3, 10), a timely question is whether and how access to TWFH modifies effects of office design on employee health. To address this question, we investigated (i) the main effects of office design and access to TWFH on sickness absence and (ii) the moderating effect of access to TWFH on the relationship between office design and sickness absence.

Sickness absence results in significant costs for individuals, organizations, and welfare states (11, 12), and we need to identify risk and protective factors to reduce these costs. Differences in experiences of indoor environmental quality (13, 14), psychological privacy (13, 15), psychosocial work factors (16, 17), and health (2, 4) across office designs indicate that this might be a relevant factor for sickness absence among office workers. To our knowledge, only five primary studies have investigated the relationship between office design and sickness absence (8). Of these, two studies found higher risk of sickness absence among employees in open-plan offices compared to employees in private offices (18, 19), and another found a significant relationship between number of sickness absence days and number of office occupants (20). The two remaining studies, however, found no consistent differences in sickness absence across office designs (21, 22). Results have also varied within each study depending on how sickness absence was measured. In one study (18), employees in shared-room and open-plan offices had significantly higher risks of sickness absence, but did not significantly differ from employees in private offices in terms of number of sickness absence days. In another study (19), employees in open-plan offices had higher risk of short-term sickness absence but not long-term sickness absence.

Moreover, effects might not be the same across all shared-room and open-plan offices (6) and findings seem particularly equivocal in light of studies that have investigated non-territorial designs. While one might expect these designs to impact health and well-being, for instance by limiting opportunities for personalization of workspace and psychological privacy (7, 23–25), previous studies on non-territorial designs and sickness absence have been inconsistent. One recent study found significantly higher sickness absence rates among employees in offices with flexible seating (22), while an earlier study found significant effects only among men (19). One study has even found significantly lower rates compared to private offices (21). More research is clearly needed on these designs. Thus, non-territorial shared-room and open-plan offices were differentiated as separate office design categories in the current study.

While previous research suggests that sickness absence might differ across office designs, evidence of an overall effect of office design on sickness absence is ambiguous and mixed. One likely reason for this inconclusiveness is that existing studies have not considered other aspects of contemporary office work as covariates or moderators. Access to TWFH may be one important factor to consider (1). The COVID-19 pandemic and technological development in recent decades has made TWFH accessible to many workers (3, 10, 26). Previous research on TWFH and health is scarce, and existing studies have found both positive and negative effects (5). To our knowledge, no previous studies have investigated the effects of access to TWFH on sickness absence or its moderating role in the relationship between office design and health. Although some studies have indicated that TWFH increases presenteeism (ie, working while ill) (27), which could result in an apparent decrease in sickness absence in the short-term, access to TWFH might protect against sickness absence through other mechanisms. As a form of time-spatial flexibility (28), access to TWFH may reduce sickness absence through increased decision latitude and job autonomy (29), which are among the strongest and most consistent predictors of outcomes such as sickness absence and disability retirement (30, 31). In a similar vein, access to TWFH might buffer some of the negative aspects that pervade shared-room and open-plan offices (eg, distractions, lack of privacy, crowding) by providing ways to regulate input and output privacy (32) or by reducing time spent coping with environmental stressors (33).

Based on the above reasoning and empirical findings, we expected that sickness absence would be higher among employees in conventional and non-territorial shared-room and open-plan offices than among employees in private offices. We further expected that sickness absence would be lower among employees with access to TWFH, irrespective of office design. Finally, we expected that differences in sickness absence between office designs would be less pronounced among employees with access to TWFH (ie, access to TWFH would attenuate effects of conventional and non-territorial shared-room and open-plan offices).

## Methods

### Sample and procedure

The study used cross-sectional data collected by personal telephone interviews between October 2019 and March 2020 as part of the Level of Living Survey on Working Conditions by Statistics Norway (34). Potential participants received written information by mail prior to telephone contact and participation was based on informed consent. The gross sample consisted of 19 687 individuals randomly drawn from the Norwegian population aged 17–67 years of age. Of these, 11 212 (57%) participated in the survey. Eligible participants in the current study were employees in paid work who primarily worked between 06:00–18:00 hours (ie, daytime work) and performed all or parts of their work in an office (ie, a place where business, clerical, or professional activities are conducted). Since participants reported their sickness absence retrospectively based on the last 12 months, we excluded participants who had changed employer – and likely also office concept – in 2019 or 2020. The final sample consisted of 4329 participants, after excluding participants with missing data on any of the focal variables (ie, office design, access to TWFH, sickness absence).

### Office concept variables

In keeping with a three-dimensional definition of office concepts (1), we created a categorical variable for *office design* based on office layout (“Do you work in your own office, shared-room office, or office landscape?”) and whether seating was fixed or free (“Do you have a fixed workstation?”). The latter distinguished between shared-room and open-plan offices with fixed seating (ie, conventional shared-room and open-plan offices) and shared-room and open-plan offices with shared seating (ie, non-territorial shared-room and open-plan offices). Non-territorial shared-room and open-plan offices were merged to ensure enough observations in all groups. This resulted in four office categories (ie, private, conventional shared-room, conventional open-plan, and non-territorial offices; see table 1). *Access to TWFH* was measured by a dichotomous variable based the question “Can you work from home in your current job?”, to which participants responded “yes” or “no”.

**Table 1:** Office design categories and definitions in the current study.

Office design category	Main characteristics
Private office	Office room occupied by one employee; fixed seating.
Conventional shared-room office	Office room occupied by several employees; fixed seating.
Conventional open-plan office	Office landscape occupied by several employees; fixed seating.
Non-territorial office	Office room or office landscape occupied by several employees; free seating.

### Sickness absence

In Norway, employees can self-certify their own sickness absence according to one of two regimes, depending on whether their employer follows the general rules for sickness absence or is part of the agreement between the Norwegian Government and Social Partners on a More Inclusive Working Life (the IA Agreement). The general rules for sickness absence permit employees to self-certify four times each year for up to three consecutive days, while the IA Agreement permits employees to self-certify 24 days in total during a 12-month period, where each spell can last up to 8 days. The current study used a self-reported measure of the number of *self-certified sickness absence* episodes in the last 12 months based on two questions: (i) “Have you had self-certified sickness absence in the last 12 months?”, and (ii) “How many times/episodes with self-certified sickness absence have you had in the last 12 months?”.

### Statistical analysis

Statistical analyses were performed in R 4.2.1 (35). To incorporate excess zeros and over-dispersion, we used negative binomial hurdle models with the hurdle() function from the pscl package (36, 37). A logistic regression component for zero versus larger counts (ie, a zero hurdle model) estimated odds ratios (OR) for having had at least one sickness absence episode during the last 12 months, while a zero-truncated negative binomial component (ie, a count data model) estimated incidence rate ratios (IRR) for the number of sickness absence episodes among participants with sickness absence. The unadjusted model (model 1) included the predictor variables office design and access to TWFH. Demographic characteristics may impact sickness absence and have been found to differ between office designs (6). Thus, adjusted models (model 2) included the predictor variables, and the covariates age (mean-centered), gender, education level (ie, primary/lower secondary school, upper secondary school, university/college 1–4 years, university/college ≥5 years), a dichotomous variable for leadership responsibility (“does your position include leadership responsibility so that other people work under your supervision, or is it otherwise an executive position?”), and a dichotomous variable for how much time the participant spent on office work (ie, ≥50% of the time or <50%). To test for moderating effects of access to TWFH, model 3 included a multiplicative term between office design and access to TWFH in addition to all the above variables. Due to the inherent nonlinearity in generalized linear models, interpretation of interaction effects is complex and may lead to incorrect conclusions if based only on interaction term coefficients (38). The current study used chi-squared tests to test for moderation (ie, omnibus tests of interaction effects) and estimated marginal means (ie, predicted probabilities for the zero hurdle model and predicted mean counts for the count data model) for pairwise comparisons in post-hoc analyses (performed with the emmeans R package). Statistical significance was determined by 95% confidence intervals (CI) or a significance level of P<0.05.

## Results

### Descriptive statistics

Table 2 displays sample characteristics, both overall and by office design. Private office was most common (45%), followed by conventional open-plan (27%), conventional shared-room (18%), and non-territorial (9%) offices. Mean age was around 46 [standard deviation (SD) 11.13] years and 46% were women. Demographic (ie, age, gender, education level) and work-related characteristics (ie, leadership responsibility, time spent doing office work) differed across office designs, confirming the need to control for these in subsequent analyses. Access to TWFH also differed across office designs; from 47% in non-territorial offices to 76% in conventional open-plan offices. Altogether, 48% reported having had self-certified sickness absence. Average number of episodes with self-certified sickness absence was 1.03 (SD 1.61; median=0; range=0–24).

**Table 2:** Sample characteristics, overall and by office design. [SD=standard deviation.]

Variable	Overall sample (N=4329)		Private office (N=1982)		Shared-room office (N=788)		Open-plan office (N=1171)		Non-territorial office (N=388)		Office design differences ^a^
					
	Mean (SD)	N (%)	Mean (SD)	N (%)	Mean (SD)	N (%)	Mean (SD)	N (%)	Mean (SD)	N (%)	P-value
Age	45.97 (11.13)		48.22 (10.31)		44.86 (11.43)		44.54 (11.07)		41.05 (12.11)		<0.001
Gender											<0.001
Female		2008 (46)		852 (43)		407 (52)		563 (48)		186 (48)	
Male		2321 (54)		1130 (57)		381 (48)		608 (52)		202 (52)	
Educational level											<0.001
University/college ≥5 years		1018 (24)		469 (24)		165 (21)		318 (27)		66 (17)	
University/college 1–4 years		1685 (39)		709 (36)		340 (43)		495 (42)		141 (36)	
Upper secondary school		1139 (26)		579 (29)		196 (25)		234 (20)		130 (34)	
Primary/lower secondary school		479 (11)		223 (11)		84 (11)		122 (10)		50 (13)	
Missing		8		2		3		2		1	
Leadership responsibility											<0.001
No		2425 (58)		862 (46)		492 (63)		817 (70)		254 (66)	
Yes		1768 (42)		999 (54)		287 (37)		351 (30)		131 (34)	
Missing		136		121		9		3		3	
Time spent doing office work											<0.001
≥50%		3262 (75)		1656 (84)		423 (54)		984 (84)		199 (51)	
<50%		1067 (25)		326 (16)		365 (46)		187 (16)		189 (49)	
Access to telework from home											<0.001
No		1386 (32)		563 (28)		335 (43)		283 (24)		205 (53)	
Yes		2943 (68)		1419 (72)		453 (57)		888 (76)		183 (47)	
Self-certified sickness absence											
≥1 episode		2083 (48)		828 (42)		395 (50)		654 (56)		206 (53)	<0.001
Number of episodes	1.03 (1.61)		0.84 (1.47)		1.14 (1.67)		1.20 (1.65)		1.28 (1.95)		<0.001

a Test of difference between office designs: Kruskal-Wallis rank sum test; Pearson’s Chi-squared test

### Main effects of office design and access to telework from home

Table 3 displays findings for the main effects of office design and access to TWFH on self-certified sickness absence. Unadjusted models (model 1) indicated that when compared to employees in private offices, employees in conventional shared-room (OR 1.36, 95% CI 1.16–1.61), conventional open-plan (OR 1.78, 95% CI 1.54–2.06), and non-territorial (OR 1.51, 95% CI 1.21–1.88) offices had significantly higher odds of having had ≥1 episode of sickness absence. Unadjusted models also indicated significantly higher rates of sickness absence (ie, number of episodes) among employees in conventional shared-room (IRR 1.25, 95% CI 1.06–1.48) and non-territorial (IRR 1.37, 95% CI 1.11–1.69) offices compared to employees in private offices. The effect of conventional open-plan offices in the zero hurdle model (OR 1.32, 95% CI 1.13–1.54) remained statistically significant in the adjusted model (model 2). Post-hoc analyses indicated that, in addition to the significant difference between private offices and conventional open-plan offices, the predicted probability of self-certified sickness absence was significantly lower in conventional shared-room offices than in conventional open-plan offices (*z*=-2.25, P=0.025). There were no significant differences between non-territorial offices and conventional shared-room (*z*=0.60, P=0.551) or conventional open-plan (*z*=1.16, P=0.246) offices (see supplementary material, www.sjweh.fi/article/4078).

**Table 3:** Effects of office design and access to telework from home on self-certified sickness absence. [OR=odd ratio; CI=confidence interval; IRR=incidence rate ratio; TWFH=telework from home.] **Statistically significant coefficients in bold**.

	Model 1 ^a^		Model 2 ^b^		Model 3 ^b^	
		
	OR (95% CI)	IRR (95% CI)	OR (95% CI)	IRR (95% CI)	OR (95% CI)	IRR (95% CI)
Zero hurdle model						
Conventional shared-room office ^c^	**1.36**(1.16–1.61)		1.06 (0.88–1.27)		0.96 (0.72–1.28)	
Conventional open-plan office ^c^	**1.78** (1.54–2.06)		**1.32** (1.13–1.54)		0.91 (0.68–1.23)	
Non-territorial office ^c^	**1.51** (1.21–1.88)		1.14 (0.90–1.44)		1.08 (0.76–1.52)	
Access to telework from home	**0.83** (0.73–0.94)		**0.86** (0.74–0.99)		**0.74** (0.60–0.91)	
Conventional shared-room office × access to TWFH					1.14 (0.80–1.63)	
Conventional open-plan office × access to TWFH					**1.63** (1.16–2.30)	
Non-territorial office × access to TWFH					1.04 (0.65–1.65)	
Count data model						
Conventional shared-room office ^c^		**1.25** (1.06–1.48)		1.05 0.88–1.25)		0.97 (0.74–1.26)
Conventional open-plan office ^c^		1.15 (0.99–1.33)		1.02 (0.88–1.17)		1.17 (0.90–1.53)
Non-territorial office ^c^		**1.37 (**1.11–1.69)		1.17 (0.95–1.44)		1.15 (0.85–1.54)
Access to TWFH		0.91 (0.80–1.04)		1.01 (0.89–1.15)		1.03 (0.84–1.26)
Conventional shared-room office × access to TWFH						1.16 (0.83–1.61)
Conventional open-plan office × access to TWFH						0.82 (0.60–1.12)
Non-territorial office × access to TWFH						1.05 (0.69–1.59)

a Crude, unadjusted, model.

b Model adjusted for age, gender, education level, leadership responsibility, and time spent on office work.

c Reference category = private office.

Findings for a main effect of access to TWFH indicated significantly lower odds of self-certified sickness absence among employees with access to TWFH compared to those with no access (OR 0.86, 95% CI 0.74–0.99). Access to TWFH had no significant effect on the number of self-certified sickness absence episodes (IRR 1.01, 95% CI 0.89–1.15).

### Moderating effects of access to telework from home

**Figure 1:**
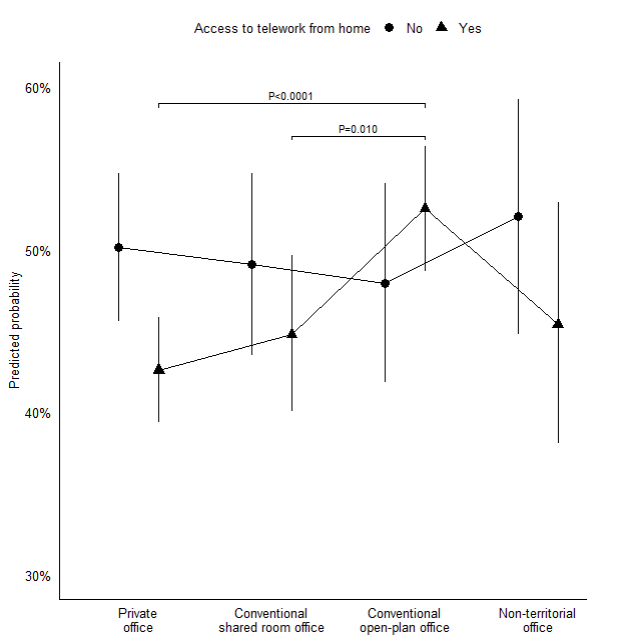
Interaction between office design and access to telework from home. Predicted probabilities and 95% confidence intervals. Results are averaged over age, gender, education level, leadership responsibility, and time spent on office work. Only significance tests within each level of the moderator and with P<0.05 are shown.

Table 3 (model 3) displays findings for the moderating effect of access to TWFH on the relationship between office design and self-certified sickness absence. An omnibus test of the interaction between office design and access to TWFH indicated that access to TWFH significantly moderated the relationship between office design and self-certified sickness absence in the zero hurdle model [*X^2^* (3)=8.15, P=0.043]. Plots of predicted probabilities of having had ≥1 day of self-certified sickness absence (figure 1) indicated that employees in conventional open-plan offices differed from employees in the other office designs in the direction of the interaction effect; while employees in private, conventional shared-room, and non-territorial offices with access to TWFH had lower probability of sickness absence than employees without access to TWFH, the opposite was the case for employees in conventional open-plan offices. Consequently, and contrary to expectations, differences between office designs were larger – rather than smaller – among employees who had access to TWFH. Specifically, among employees with access to TWFH, those in conventional open-plan offices had significantly higher predicted probability of self-certified sickness absence than those in both private (*z*=4.41, P<0.0001) and conventional shared-room (*z*=2.58, P=0.010) offices, while there were no significant differences between office designs among employees who did not have access to TWFH (see supplementary material).

An omnibus test indicated no significant interaction effects in the negative binomial hurdle model as a whole [*X^2^* (3)=11.99, P=0.062]. This was also confirmed in post-hoc analyses where pairwise comparisons indicated no significant differences in predicted mean counts (see supplementary material).

### Sensitivity analyses

Since effects might depend on how much time is spent in an office environment (ie, exposure duration), we performed separate analyses that included only employees who worked ≥30 hours per week and spent >50% of their time on office work. Results were comparable to the main analyses, and pairwise comparisons revealed no significant differences beyond those observed in the overall sample.

## Discussion

Advantages and disadvantages of contemporary office concepts is an ongoing debate in both science and society at large. Many organizations implement shared-room, open-plan, and non-territorial office designs to cut costs and boost collaboration, but negative consequences for employee health might render the net benefit unclear for both individuals, organizations, and welfare states (6). As expected, we found that employees in conventional open-plan offices had higher risk of self-certified sickness absence compared to employees in private offices. Our findings corroborate previous research that have identified open-plan offices as potential precursors of sickness absence (18–20). In contrast to some previous studies (18, 20), employees in conventional shared-room offices did not significantly differ from employees in private offices. Interestingly, post-hoc analyses indicated that employees in conventional shared-room offices had significantly lower probability of self-certified sickness absence compared to employees in conventional open-plan offices with adjusted estimates closer to employees in private offices.

Non-territorial shared-room and open-plan offices did not significantly differ from any of the other office designs. While this could indicate that non-territoriality is not in itself a risk factor for sickness absence, the findings should be interpreted with caution since our study did not distinguish between different non-territorial office types. Non-territorial offices vary greatly – from mere shared seating to activity-based offices with designated zones for different work tasks. Scholars have argued that effects of the latter might differ from those of conventional open-plan offices because different mechanisms work in opposite directions (eg, lack of personalization of space versus increased control over where to work) (23) and some empirical findings support this (6, 7). Importantly, sample characteristics in this office category do not point to activity-based working; only around half of participants had access to TWFH and a large share spent <50% of their time on office work. Thus, this group is likely very diverse, which could explain the absence of significant findings. On the other hand, results from analyses that included only employees who worked ≥30 hours per week and primarily did office work revealed no additional significant findings. Studies that distinguish between different non-territorial offices are greatly needed, particularly studies that focus on activity-based versus conventional open-plan offices.

In sum, while our findings point to interesting pairwise differences between office categories, they are inconsistent in terms of an overall effect of office design on sickness absence. This inconsistency is in line with previous findings (8) and indicates the need for more high-quality studies of the relationship between office design and sickness absence, preferably with registry data on sickness absence. This also includes research on mediators to help reveal potential mechanisms behind the observed differences between office design categories.

Most existing studies on TWFH and health have used subjective experiences as outcomes (5). The current study adds to this limited research body by investigating main effects of access to TWFH on a less subjective measure of health (ie, self-certified sickness absence). As expected, we found significantly lower risk of self-certified sickness absence among employees with access to TWFH than among employees without. Notwithstanding the possibility that the effect was due to increased presenteeism among employees with access to TWFH (27), this finding is highly relevant for post-pandemic working life. As TWFH is unlikely to return to pre-pandemic levels, many organizations are adapting to new ways of working with little evidence-based knowledge to rely on. Thus, future studies should continue this research line and investigate effects of TWFH on health outcomes relevant for organizations.

To our knowledge, this is the first study to investigate the role of TWFH in the context of office design and sickness absence. We found that access to TWFH moderated the relationship between office design and sickness absence, although not as expected; access to TWFH amplified, rather than attenuated, differences between private and conventional open-plan offices. Previous studies have indicated that effects of flexible work arrangements depend on whether the arrangement is perceived as voluntary (29, 39). One explanation for our finding could be that employees in private and open-plan offices differ in perceptions of freedom of choice in teleworking from home as a function of the office environment. If employees in open-plan offices telework from home primarily to avoid negative aspects in the office environment (eg, distractions, lack of privacy, crowding), the true flexibility and autonomy in teleworking from home may be questioned. From a time-spatial, job-crafting perspective (40), those employees in open-plan offices who involuntarily telework from home, or do so only to avoid poor working conditions in the office environment, will thus experience the demands associated with constant connectivity (41, 42) without the benefits associated with perceived time-spatial flexibility. Health effects of TWFH might also depend on employee characteristics and home facilities (5). Since demographic characteristics might differ across office concepts, such as higher seniority and pay in private offices (6), employees in private offices could likely afford better home facilities (eg, more space, separate room for home office), which may have contributed to the differences we observed.

Another explanation concerns access to TWFH as an HR policy as opposed to the actual use of TWFH by employees. Like job design and redesign in general (43), access to TWFH may lead to unintended consequences, for instance in terms of how known problems in open-plan offices are managed. Some organizations might – explicitly or implicitly – view access to TWFH as a remedy for problems in open-plan offices and devote less attention to addressing environmental stressors compared to organizations that do not allow TWFH. Thus, an important practical implication of our findings is that organizations should be wary of using TWFH as a buffer of negative effects of open-plan offices. An interesting avenue for future research is to investigate the moderating role of different organizational policies in the relationship between office concepts and health.

### Strengths and limitations

The current study used data from a large nationally representative sample of office employees with a satisfactory response rate. Thus, the external validity of the findings is one of the study’s key strengths and sets it apart from most other studies on office concepts and health, which are often characterized by data sampled at the organizational level and low sample size (6). Furthermore, few high-quality studies have investigated relationships between office design, access to TWFH, and health; even fewer have investigated how different aspects of contemporary office work interact. Considering this paucity, cross-sectional studies represent valuable contributions that can bring the field forward by indicating “whether pairs of variables are related and whether moderators might be at play” (44, p133). The cross-sectional study design nevertheless warrants caution in interpretation of results. Participants reported sickness absence retrospectively based on the last 12 months. While we only included participants who worked at their current workplace prior to the period for which they reported sickness absence, it is possible that some participants changed office design or access to TWFH during the period. Consequently, we cannot rule out reverse causation. For instance, some employees in open-plan offices might have been given access to TWFH as a consequence of sickness absence, which could have produced the pattern we observed between TWFH and sickness absence in open-plan offices. Since the current study was cross-sectional, we could not investigate reverse causality but encourage future research to do so.

There are also limitations related to which variables were included and how they were measured. Survey questions did not define what was meant by the different office categories or specify the number of people in shared-room offices. Thus, participants selected the category they found most fitting, irrespective of the number of people they shared an office with. The study is also limited in the extent to which the results can be generalized to modern offices, as it did not capture all relevant aspects of contemporary office design (eg, activity-based workspaces versus other non-territorial offices). Apart from a dichotomized variable for time spent on office work, the study also lacked information about type of office work (eg, work tasks, occupation) and work performed by participants who spent most of their time on other tasks. It also lacked information about the participants’ home office situation, including how much participants worked from home. The validity of the sickness absence measure might have been influenced by recall bias. However, while participants may have had trouble recalling the accurate number of self-certified sickness absence episodes, they are less likely to have misremembered having had sickness absence at all.

### Concluding remarks

Contemporary office work is dynamic and multifaceted, and research on office concepts should take this complexity into account. The current study extends existing research by investigating effects of office concepts on sickness absence considering aspects of both office layout (ie, private, shared-room, and open-plan offices), office use (ie, non-territoriality), and office location (ie, access to TWFH). Understanding predictors of sickness absence is important to ensure a sustainable working life. Our findings confirmed those of previous studies by identifying conventional open-plan offices as a potential risk factor for sickness absence compared to both private and conventional shared-room offices. They also indicated that while access to TWFH may protect against sickness absence overall, it might not buffer the detrimental effects of conventional open-plan offices. On the contrary, access to TWFH amplified differences in sickness absence risk between private and conventional open-plan offices. Giving employees opportunity to work in private offices is likely not a viable option for many organizations. Future studies should therefore continue to investigate mechanisms and conditions for how and when shared-room and open-plan offices might be harmful for employee health to help identify potential solutions with practical relevance for organizations.

## Supplementary material


