# Supplementary material for: Office design, telework from home, and self-certified sickness absence: A cross-sectional study of main and moderating effects in a nationally representative sample
Source: Scand J Work Environ Health. 2023 Mar 30;49(3):222–30. doi: 10.5271/sjweh.4078 (PMC10621899; doi:10.5271/sjweh.4078)
Supplement: Supplementary file 1 [file SJWEH-49-222-S001.pdf]

Office design, telework from home, and self-certified sickness absence: A cross-sectional study of main and moderating effects in a nationally representative sample[1]

by Randi Hovden Borge, MSc,[2] Håkon A Johannessen, PhD, Knut Inge Fostervold, PhD, Morten Birkeland Nielsen, PhD

1. *Supplementary material*
2. *Correspondence to: Randi Hovden Borge, National Institute of Occupational Health, Pb 5330 Majorstuen, 0304 Oslo, Norway. [E-mail: randi.borge@stami.no]*

Pairwise comparisons of estimated marginal means: Results from main effects models

Table S1. Contrasts of predicted probabilities of having at least one episode of self-certified sickness absence between office designs.[a,b]

Contrast	Estimate	SE	z ratio	p-value
Private office - Shared room office	-0.0560	0.0921	-0.607	0.5435
Private office - Open-plan office	-0.2768	0.0789	-3.507	0.0005
Private office - Non-territorial office	-0.1323	0.1201	-1.102	0.2707
Shared room office - Open-plan office	-0.2208	0.0983	-2.247	0.0247
Shared room office - Non-territorial office	-0.0764	0.1282	-0.596	0.5515
Open-plan office - Non-territorial office	0.1444	0.1245	1.160	0.2460

[a]Results are averaged over access to telework from home, age, gender, education level, leadership responsibility, and time spent on office work.
[b]Results are given on the log odds ratio (not the response) scale.

Table S2. Contrasts of predicted mean counts of having at least one episode of self-certified sickness absence between office designs.[a]

Contrast	Estimate	SE	t ratio	p-value
Private office - Shared room office	-0.0555	0.1009	-0.550	0.5823
Private office - Open-plan office	-0.0195	0.0822	-0.238	0.8122
Private office - Non-territorial office	-0.1861	0.1342	-1.387	0.1656
Shared room office - Open-plan office	0.0359	0.1023	0.351	0.7253
Shared room office - Non-territorial office	-0.1306	0.1413	-0.924	0.3553
Open-plan office - Non-territorial office	-0.1666	0.1360	-1.224	0.2209

[a]Results are averaged over access to telework from home, age, gender, education level, leadership responsibility, and time spent on office work.

Pairwise comparisons of estimated marginal means: Results from interaction effects models

Table S3. Contrasts of predicted probabilities of at least one episode of self-certified sickness absence between different office designs, by access to telework from home.[a,b]

**Access to telework from home = No**				
**Contrast**	**Estimate**	**SE**	**z ratio**	**p-value**
Shared room office - Private office	-0.0421	0.1478	-0.285	0.7756
Open-plan office - Private office	-0.0889	0.1526	-0.583	0.5601
Open-plan office - Shared room office	-0.0468	0.1683	-0.278	0.7811
Non-territorial office - Private office	0.0767	0.1761	0.436	0.6631
Non-territorial office - Shared room office	0.1189	0.1846	0.644	0.5196
Non-territorial office - Open-plan office	0.1656	0.1923	0.861	0.3890
**Access to telework from home = Yes**				
**Contrast**	**Estimate**	**SE**	**z ratio**	**p-value**
Shared room office - Private office	0.0896	0.1146	0.782	0.4341
Open-plan office - Private office	0.4005	0.0909	4.405	<.0001
Open-plan office - Shared room office	0.3108	0.1205	2.580	0.0099
Non-territorial office - Private office	0.1141	0.1627	0.701	0.4831
Non-territorial office - Shared room office	0.1141	0.1795	0.136	0.8916
Non-territorial office - Open-plan office	-0.2864	0.1667	0.136	0.0859

[a]Results are averaged over access to telework from home, age, gender, education level, leadership responsibility, and time spent on office work.
[b]Results are given on the log odds ratio (not the response) scale.

Table S4. Contrasts of predicted mean counts of self-certified sickness absence episodes between office designs, by access to telework from home.[a]

**Access to telework from home = No**				
**Contrast**	**Estimate**	**SE**	**t ratio**	**p-value**
Shared room office - Private office	-0.0378	0.1472	-0.257	0.7973
Open-plan office - Private office	0.1920	0.1641	1.170	0.2419
Open-plan office - Shared room office	0.2299	0.1755	1.310	0.1903
Non-territorial office - Private office	0.1610	0.1836	0.877	0.3805
Non-territorial office - Shared room office	0.1988	0.1856	1.071	0.2842
Non-territorial office - Open-plan office	-0.0310	0.2060	-0.151	0.8802
**Access to telework from home = Yes**				
**Contrast**	**Estimate**	**SE**	**t ratio**	**p-value**
Shared room office - Private office	0.1329	0.1344	0.989	0.3229
Open-plan office - Private office	-0.0379	0.0961	-0.394	0.6936
Open-plan office - Shared room office	-0.1707	0.1325	-1.289	0.1976
Non-territorial office - Private office	0.2309	0.2002	1.153	0.2488
Non-territorial office - Shared room office	0.0980	0.2197	0.446	0.6556
Non-territorial office - Open-plan office	0.2688	0.2000	1.344	0.1791

[a]Results are averaged over access to telework from home, age, gender, education level, leadership responsibility, and time spent on office work.
